# Applicability of PolyActive™ Thin Film Composite Membranes for CO_2_ Separation from C_2_H_4_ Containing Multi-Component Gas Mixtures at Pressures up to 30 Bar

**DOI:** 10.3390/membranes8020027

**Published:** 2018-06-05

**Authors:** Karina Schuldt, Jan Pohlmann, Sergey Shishatskiy, Torsten Brinkmann

**Affiliations:** Helmholtz-Zentrum Geesthacht, Institute of Polymer Research, Max-Planck-Str. 1, 21502 Geesthacht, Germany; karina.schuldt@hzg.de (K.S.); jan.pohlmann@hzg.de (J.P.); sergey.shishatskiy@hzg.de (S.S.)

**Keywords:** gas permeation, CO_2_ separation, PolyActive™, thin film composite membrane, oxidative coupling of methane

## Abstract

The PolyActive™ thin film composite membrane (TFCM) has already been successfully applied for CO_2_ separation tasks at feed pressures up to 10 bar. To investigate the applicability at higher pressures, measurements were undertaken with C_2_H_4_ containing gas mixtures with a composition comparable to the product stream of the oxidative coupling of methane process, as well as single gases up to a feed pressure of 30 bar. Furthermore, the permeances of the conducted gas mixture experiments were simulated. The results show a strong swelling influence of CO_2_ on the used membrane depending on the CO_2_ fugacity. This swelling effect leads to a pronounced decrease in selectivity. The observed membrane behavior at high pressures could not be predicted by the Free Volume Model (FVM). Two different simulations were conducted: one based on parameters calculated from single gas data measured at pressures up to 2 bar; and a second based on parameters calculated from single gas data measured at pressures from 2 to 30 bar. The two simulations differ in their prediction accuracy. However, they confirm that it is possible to predict the measured permeances in the pressure range up to an average CO_2_ fugacity of 6 bar.

## 1. Introduction

Membrane processes are constantly becoming more significant in the field of gas separation. A requirement for the introduction of a membrane process is a reliable membrane material, which preferably has a high gas selectivity as well as a high permeability. Those membrane materials can be implemented as selective layers of composite membranes installed in a membrane module. Furthermore, simulation tools capable of describing the behavior of every component in the system with appropriate accuracy are necessary. For CO_2_ separation from gaseous mixtures, the Institute of Polymer Research of Helmholtz-Zentrum Geesthacht (HZG) developed a thin film composite membrane (TFCM) with the polyethylene oxide containing block copolymer PolyActive™ as selective layer [1,2]. A remarkable feature of this membrane is the high CO_2_/N_2_ selectivity at relatively high CO_2_ permeance, which are favorable characteristics for an efficient membrane application [3,4]. Experiments at laboratory and pilot-plant scale have proven the good CO_2_ separation characteristics of this membrane at pressures up to 10 bar [5]. Nevertheless, there are several processes, which require the separation of CO_2_ from permanent gases and C_1_–C_3_ hydrocarbons at higher pressures. One of those processes is the separation of CO_2_ from a C_2_H_4_ containing multi-component gas mixture. This separation task occurs in the purification step after the oxidative coupling of methane, which is investigated on pilot scale as an attractive alternative to conventional steam reforming for the production of C_2_H_4_, and contains CO_2_ as a byproduct [6]. To make membrane processes competitive to conventional separation processes of, for example, chemical absorption using amines [7], a well performing membrane is necessary. Additionally, a reliable model to predict the permeances has to be developed. At pressures up to 10 bar, the Free Volume Model (FVM) developed by Fang et al. [8] has already been successfully applied for CO_2_ separation from various gas mixtures with PolyActive™ TFCM [5], and for modeling of hydrocarbon separation [9]. The FVM has the advantage that parameters required for the description of mixed gas separation can be determined from single gas experiments. Investigations with gas mixtures similar to the product stream of the oxidative coupling of methane reaction were carried out at Technische Universität Berlin. The experimental and simulation results for a mini plant module were compared for pilot plant investigations at 10 and 20 bar feed pressures [5]. The results indicated that the FVM used for the calculation of the permeances for the multi-component permeation does not adequately consider the effect of membrane swelling at the higher pressures, i.e., 20 bar. Single gas data at low pressures were used to determine the FVM parameters. To take into consideration the selected data set from which the model parameters were determined as influencing factors for the precision of the prediction, single gas experiments were done at low and at high pressures. Hence, the corresponding FVM parameters and the resulting simulated data, depending on different data sets, are compared in this publication. The major aim of the current work is to investigate the swelling influence of CO_2_ on the PolyActive™ TFC membrane and its predictability at pressures up to 30 bar by varying the CO_2_ fugacity in the feed stream. Furthermore, the impact of a second swelling component, i.e., C_2_H_4_, in the mixture on the separation performance was assessed, since most of the investigation using this type of membrane were conducted using mixtures of CO_2_ and non-swelling components such as N_2_ or CH_4_.

## 2. Materials and Methods

### 2.1. Gases

The gases used in the single and mixed gas permeation experiments had a purity of at least 99.5%. CO_2_ and CH_4_ were purchased from Linde, while C_2_H_4_ and N_2_ were purchased from Air Liquide.

### 2.2. Membrane Material

**Figure 1:**
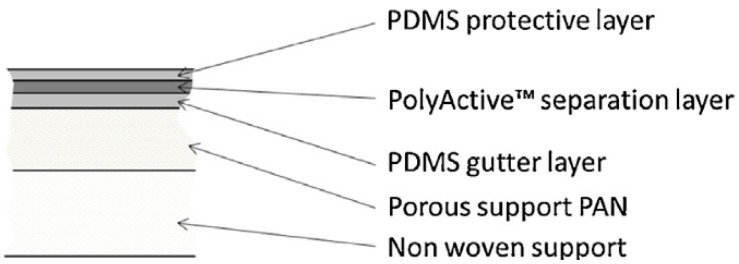
Schematic representation of the cross-sectional structure of the multilayer thin film composite membrane [11].

The TFC membrane used in this study is a multilayer TFC membrane with PolyActive™ 1500 as the selective layer. The selective layer with a thickness of about 70 nm was made of poly (ethylene oxide) (PEO) soft segments (77 wt %) and poly (butylene terephthalate) (PBT) hard segments (23 wt %) [1]. Furthermore, the multilayer membrane contains a protective layer as well as a gutter layer between separation layer and porous polyacrylonitrile (PAN) support layer. Polydimethylsiloxane was used as a material for these two layers. The layers are sequentially formed on a support structure of non-woven polyester. The schematic structure of the multilayer PolyActive™ TFCM is shown in Figure 1. The membrane investigated in the current study was taken from a pilot scale membrane batch as regularly produced with membrane casting and coating machines developed and operated at HZG [1,2,10].

Gas separation experiments were carried out using membrane samples with a CO_2_ permeance of about 4 m³_STP_ m^−^² h^−1^ bar^−1^ (1 m³_STP_ m^−^² h^−1^ bar^−1^ = 370.3 GPU = 1.248 × 10^−7^ mol m^−2^ s^−1^ Pa^−1^) at standard conditions (p = 1.01325 bar, T = 273.15 K) and a CO_2_/N_2_ selectivity of 55 determined in single gas laboratory tests at room temperature. These values are inside the characteristic range for PolyActive™ thin film composite membranes at ambient conditions (p = 1.01325 bar, T = 273.15 K) [2]. 

**Figure 2:**
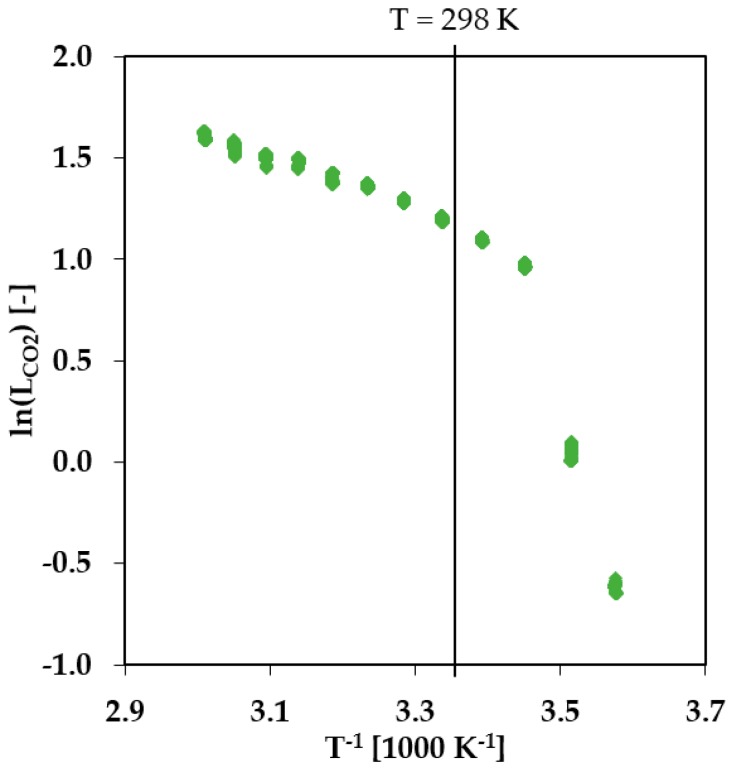
CO_2_ single gas transport experiment with PolyActive™ thin film composite membrane at different temperatures. Temperature range 5–60 °C, feed pressure 200–1200 mbar, permeate pressure 0–10 mbar.

An important parameter for the transport of gases through polymer membranes is the state of the polymer in the selective layer, which depends on the temperature of the system. While PBT is in a semicrystalline state at any conditions typical for a practical gas separation process, PEO with a molecular weight of 1500 g/mol is in the semicrystalline state only at sub-ambient temperatures. An amorphous state of PEO was intended, so that the PEO block performs as the primary phase for the gas transport with characteristics typical for rubbery polymers [12]. To prove whether the membrane material is in a rubbery, glassy, or semicrystalline state at the chosen experimental conditions of 25 °C, single gas transport measurements at different temperatures have been carried out using a fully automated constant volume-variable pressure apparatus. The experimental setup, as well as the functional principle, are described in more detail in [1,9]. The results for the CO_2_ permeance in a temperature range 5–60 °C are presented in Figure 2. As can be seen from Figure 2, during the stepwise heating of the membrane, the permeance of the membrane changes drastically and fully reversibly [13] at a temperature of app. 15 °C. The PEO phase of the PolyActive™ thin selective layer changes from the semicrystalline state to the amorphous/rubbery state. At temperatures above 15 °C, the gas transport properties of the membrane show an Arrhenius dependency. This experiment demonstrates that at temperatures above 25 °C, the PEO phase of the PolyActive™ 1500 in the TFCM is in an amorphous state. Accordingly, any effect related to crystallization of the PEO block in the selective layer can be disregarded in this study.

### 2.3. Free Volume Model

The design of a membrane separation process requires a well-performing model describing the mass transfer through the membrane over a range of operating conditions which is as wide as possible. Particularly cost and time saving are models that are based on single gas transport data obtained using lab scale equipment. The FVM allows the estimation of the permeances of the components of a gas mixture depending on parameters determined from single gas experiments (described in Section 2.4). The model is based on the solution-diffusion model and on the assumption that the selective layer of the membrane is composed of a dense, defect-free material. Previous studies demonstrated that it is possible to predict the permeances in mixed gas experiments for PolyActive™ TFC membranes at feed pressures up to 10 bar with parameters derived from single gas experimental data at feed pressures up to 2 bar [5]. 

In this study, mixed gas experiments were carried out at a constant temperature: thus, the temperature independent version of the FVM was used in this work. This version of the model, given in Equation (1), includes the parameters: $L_{i}^{0}$, which describes the permeance for the average fugacity $f_{av,j}$ approaching zero, and $m_{i}$, describing the swelling influence of the gases. These parameters are determined from the results of single gas experiments. The values for the Lennard-Jones molecule diameter $\sigma_{i}$, however, are taken from literature [14].

(1) $$L_{i}=L_{i}^{0}\cdot \exp\left(\overset{n}{\underset{j=1}{\sum }}{\left(\frac{\sigma_{i}}{\sigma_{j}}\right)}^{2}\cdot m_{j}\cdot f_{av,j}\right)$$

where $L_{i}$ represents the permeance for the component i. 

The average fugacity $f_{av,j}$ includes the experimental conditions such as gas composition $y_{j}$, pressure p, and influence of real gas behavior $\varphi_{j}$ on the feed and permeate side of the membrane. The subscript F indicates the feed side and the subscript P the permeate side. The fugacity coefficients were determined using the Soave-Redlich-Kwong equation of state as implemented in Aspen Properties V8.8^®^ [15].

(2) $$f_{av,j}=\frac{p_{F}\cdot y_{F,j}\cdot \varphi_{F,j}+p_{P}\cdot y_{P,j}\cdot \varphi_{P,j}}{2}$$

Since a TFCM is investigated in this study, it is assumed that the FVM reflects the operating performance of the entire membrane, and not solely the PolyActive^®^ layer. For the investigated operating ranges, it is assumed that PolyActive^®^ governs the separation performance. 

### 2.4. Single Gas Experiments

For the determination of the Free Volume Model parameters, single gas transport experiments were carried out with each individual component of the multi-component gas mixture. A pressure increase facility was employed for the low feed pressure experiments. This facility was also used for the single gas experiments described in Section 2.2. The facility was designed for measurements at constant temperature and varying transmembrane pressures. The applied feed pressures were between 0.14 and 1.15 bar for CO_2_ and C_2_H_4_, and 0.14 and 0.95 bar for CH_4_ and N_2_. All measurements were conducted at a temperature comparable to the temperature used for the mixed gas experiments. The experimental setup, as well as the measurement method, are described in more detail in [10].

**Figure 3:**
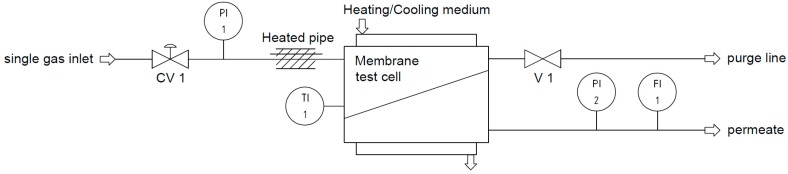
Experimental setup for single gas experiments at high pressures and constant temperature (CV: control valve, FI: flow indicator, PI: pressure indicator, TI: temperature indicator, V: valve).

For the high-pressure experiments, a simple experimental setup with a temperature controlled test cell was used, as schematically shown in Figure 3. Corresponding to the experimental conditions of the mixed gas experiments, the permeate flow rate was determined at a constant temperature of 25 °C in an absolute feed-pressure range of 2 to 30 bar in 2 bar steps. The membrane sample was the same as the one used for the low-pressure experiments. The permeance was calculated using the following equation:

(3) $$L_{i}=\frac{{\dot{V}}_{P,i,\left(STP\right)}}{A\cdot \left(f_{F.i}-f_{P,i}\right)}$$

where L represents the permeance, ${\dot{V}}_{P}$ is the volumetric flowrate of the permeate, A is the membrane area, $f_{F}$ and $f_{P}$ are fugacities of feed and permeate. 

The results can be used to calculate the gas permeance depending on the driving force. With the results for the permeance $L_{i}$, the FVM parameters were determined depending on the average fugacity $f_{av,i}$. The swelling parameter $m_{i}$ results from the slope of the experimental permeance vs. average fugacity curve, and the permeance $L_{i}^{0}$ for pressure approaching zero results from the y-axis intercept of same curve (see Equation (1)).

### 2.5. Mixed Gas Experiments

**Figure 4:**
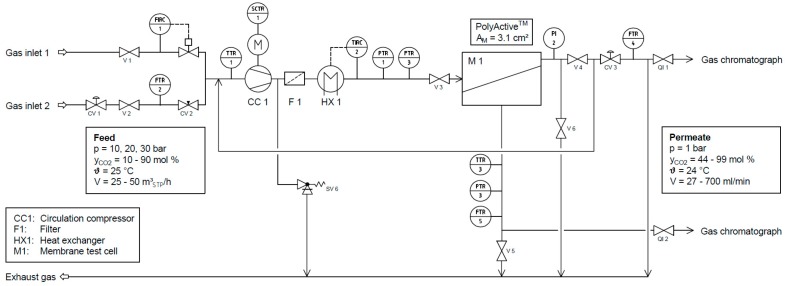
Flowsheet of experimental facility for gas separation experiments up to 30 bar at constant temperatures (CV: control valve, FI: flow indicator, FIRC: flow indicating recording controller, FTR: flow transmitting recorder, PI: pressure indicator, PTR: pressure transmitting recorder, QI: quality indicator, SCTR: speed controlling transmitting recorder, SV: safety valve, TI: temperature indicator, TIRC: temperature indicating recording controller, TTR: temperature transmitting recorder, V: valve).

The flowsheet of the laboratory scale experimental facility used to determine the mixed gas permeation behavior of the membrane is shown in Figure 4. The feed stream is supplied by two gas inlets, connected to V1 and V2, which enable the testing of different gas mixtures and the variation of the mixed gas composition while the unit is running. The most permeable gas is fed into the system through one gas inlet, while the other components making up the gas mixture are supplied as a mixture through the other inlet. This mixture is fed into a loop, which contains a dry running circulation pump (CC1), a filter (F1) and a heat exchanger (HX 1). The gas then enters a membrane test cell (M1) designed for mixed gas experiments with circular membrane samples of 47 mm in diameter. To avoid contamination of the membrane with graphite particles stemming from the vanes of the gas circulator, a filter (F1) is installed immediately downstream of the circulator. Afterwards, the gas is cooled upstream of the membrane test cell (M1) by a heat exchanger (HX1) in order to remove the heat of compression and to stabilize the experimental conditions. A mechanical backpressure regulator (CV 3) maintains the feed pressure inside the plant.

The membrane separates the feed stream into a permeate stream and a retentate stream. The majority of the retentate stream is recycled to be mixed with the feed stream. Just a small bleed stream is discharged in order to keep the less permeable components from accumulating on the feed side. This method of operation ensures low gas consumption, while at the same time maintaining a flowrate which is high enough to avoid any significant concentration polarization on the feed side of the membrane. The gas lost is that of the permeate stream and the small bleed stream. 

In order to work with the highly permeable PolyActive™ membrane at as low a stage cut as possible, the membrane surface was reduced using a reduction ring, resulting in a membrane area of 3.1 cm² for contact with the feed gas stream. The thickness of the Viton^®^ O-ring and of the O-ring holder was minimized to avoid the formation of stagnant flow zones.

The experiments were conducted at a constant temperature of 25 °C and at the three different feed pressures of 10, 20 and 30 bar. Since the influence of CO_2_ partial pressure on the membrane performance was intended to be investigated during the mixed gas experiments, the CO_2_ mole fraction in the feed gas mixture was changed from 10 to 90 mol % in 10 mol % steps. Therefore, one gas inlet was connected to a CO_2_ gas supply. The second gas inlet was connected to a gas cylinder containing a C_2_H_4_, CH_4_, and N_2_ gas mixture, which was prepared using the following procedure: the gas cylinder used for the preparation of the gas mixture was carefully evacuated; afterwards, it was filled with the pure gases up to a certain pressure, to obtain the desired composition of 22.3 mol % C_2_H_4_, 27.1 mol % CH_4_ and 50.6 mol % N_2_. The exact composition of the feed stream was determined by gas chromatographic analysis. These were conducted with an Agilent 7820 A gas chromatograph on an HP-Plot Q column. Thus, compositions similar to the product stream of the oxidative coupling of methane process could be achieved. Likewise, the composition of the bleed stream, as well as of the permeate stream, were analyzed with a gas chromatograph. The stability of experimental conditions was ensured by feeding a stream with a constant flowrate and composition into the system. The pressure in the loop was kept constant by a mechanical back-pressure regulator. In order to achieve a closed material balance and steady state conditions, the fraction of the feed stream which does not permeate the membrane leaves the system via the bleed stream. The feed flowmeter FTR 3 has a maximum flowrate of 30 m³_STP_ h^−1^ (N_2_) and was operated close to that value in all experiments. With permeate flowrates between 0.002 and 0.054 m³_STP_ h^−1^ during the conducted experiments, the stage cut was smaller than 0.2% at all experimental conditions. Due to this low stage cut and the stability of the feed gas mixture, it was safe to assume that feed and retentate compositions were essentially identical. From a combination of the measured values for permeate flowrate, as well as pressure, temperature, and composition on the feed and permeate sides, the permeance can be determined. The permeate gas flow rate was measured with a positive displacement flow meter (Bios DryCal Definer 220, MesaLabs Inc., Lakewood, CO, USA). The amount of permeate and its composition was measured when steady-state conditions were achieved. The permeance was calculated by Equation (3).

## 3. Results and Discussion

### 3.1. Determination of FVM Parameters Based on Results from Single Gas Experiments

The data sets for the determination of the FVM parameters were obtained from single gas transport experiments carried out at low and high pressures. For both cases, FVM parameters were determined and are summarized in Table 1. These FVM parameters were used for the calculation of the permeances in gas mixtures.

**Figure 5:**
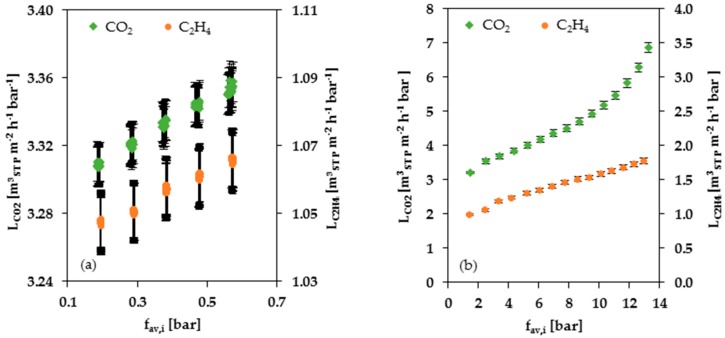
Single gas permeances of CO_2_ and C_2_H_4_ depending on the average fugacity of the particular gas at 25 °C: (**a**) pressure range of 0.14 to 1.15 bar; (**b**) pressure range of 2 to 30 bar.

Figure 5a shows CO_2_ and C_2_H_4_ permeances as a function of average fugacity obtained from single gas experiments at low pressure. For comparison, Figure 5b shows the corresponding data obtained from high pressure experiments. The permeance of CO_2_ and C_2_H_4_, as of all highly soluble gases and vapors in rubbery polymers, increases with increasing fugacity. The determined swelling parameter $m_{C_{2}H_{4}}$ for C_2_H_4_ is similar for the low- and high-pressure ranges. The swelling parameter $m_{{\mathrm{CO}}_{2}}$ for CO_2_, on the contrary, seems to differ depending on the selected data set. The CO_2_ swelling parameter obtained from high pressure experimental results is bigger than that obtained from measurements at low pressure. Furthermore, the curvature of the CO_2_ permeance curve corresponding to the single gas measurements at 2 to 30 bar (see Figure 5b) indicates that, above the average fugacity at 8 bar, the curve loses linearity and the slope increases with rising average fugacity, indicating a promoted swelling of the soft block.

In both pressure ranges, the permanent gases like CH_4_ and N_2_ showed no pressure dependency. Hence, the swelling parameter $m_{i}$ is zero for CH_4_ and N_2_. For these gases, the average value of the single gas permeance $L_{i,average}$ was determined instead of the parameters $L_{i}^{0}$ (permeance for the average fugacity $f_{av,j}$ approaching zero) and $m_{i}$ (swelling parameter). 

### 3.2. Mixed Gas Experiments and Comparison with Modeled Data

**Figure 6:**
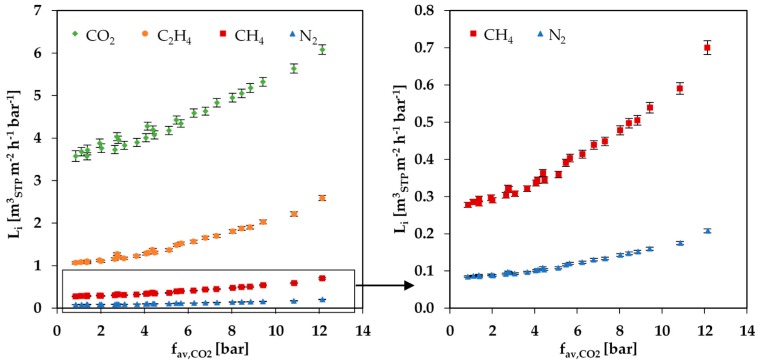
Permeances of gas mixture components depending on the average CO_2_ fugacity at 25 °C for measurement series at feed pressures of 10, 20 and 30 bar.

The experimental results for the permeance of the gas mixture experiments are summarized in Figure 6. The Figure shows the permeance of the gas mixture components plotted against the average fugacity of CO_2_. All components show an increase in permeance with an increase in average CO_2_ fugacity. In the range 1 to 12 bar of $f_{av,{\mathrm{CO}}_{2}}$ the permeances of all components except CO_2_ increase around 2.5 times, whereas the permeance of CO_2_ increases only 1.7 times. The fact that the CO_2_ fugacity has such a strong influence on the permeances of the other gases proves the strong swelling influence of CO_2_ on the PolyActive™ membrane. 

**Figure 7:**
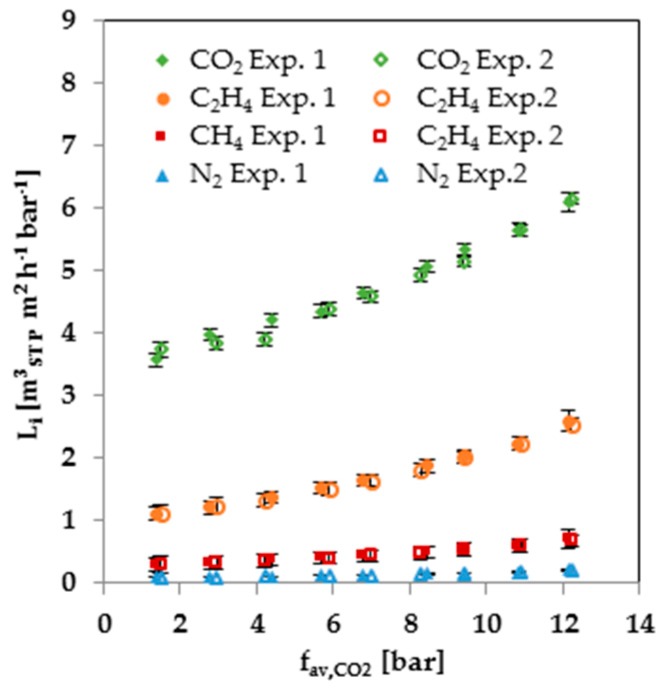
Comparison of two 30-bar mixed gas measurement series at 25 °C.

In order to prove the reproducibility of the mixed-gas measurements, the 30-bar measurement series was repeated. The results for the first and second 30-bar measurement series are shown in Figure 7. The error bars were calculated by Gaussian error propagation, which considers the influence of the errors in the measurement of each sensor on the calculated permeance. The deviation between the first and second measurement series is inside the error bars. Therefore, the results of the gas mixture experiments are reproducible. Furthermore, the repetition of the measurement shows that the swelling of the membrane is reversible.

**Figure 8:**
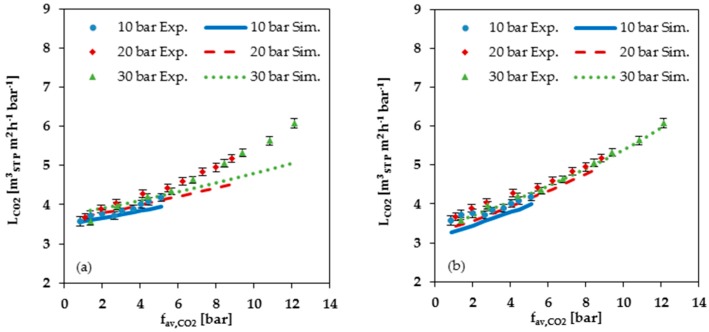
Comparison of measured data (Exp.) with simulated data (Sim.) for CO_2_ permeance at 25 °C plotted against average CO_2_ fugacity: (**a**) Simulation data based on FVM parameters determined from low pressure (up to 2 bar) single gas data; (**b**) Simulation data based on FVM parameters determined from high pressure (2 to 30 bar) single gas data.

For all components in the gas mixture, no significant difference for the measured curves in dependence of the three different feed pressures of 10, 20 and 30 bar was observed (see Figure 6). Hence, no significant dependence of the permeance on the total feed pressure could be determined. The curves of the simulated data, on the contrary, show a small difference for the three feed pressures (see Figure 8a), caused by the two-dimensional representation that does not allow for the visualization of the influence of swelling by C_2_H_4_. It is important to note that the variations in the simulated data for 10, 20 and 30 bar are almost inside the error range of the measured data.

The measured CO_2_ permeances show a small exponential increase with increasing CO_2_ fugacity for all three feed pressures (see Figure 8). The prediction of the permeance depends on the selected data set for the FVM parameters determination (see Table 1). The simulation based on single gas data at low pressures up to 2 bar shows a good prediction for smaller CO_2_ fugacities. With increasing CO_2_ fugacity, the underestimation by the FVM becomes more pronounced. The simulation based on parameters determined from single gas data recorded at higher pressures up to 30 bar shows less accurate prediction at low pressures, but resulted in a more accurate prediction of the shape of the curve via the entire investigated pressure range (see Figure 8b).

**Figure 9:**
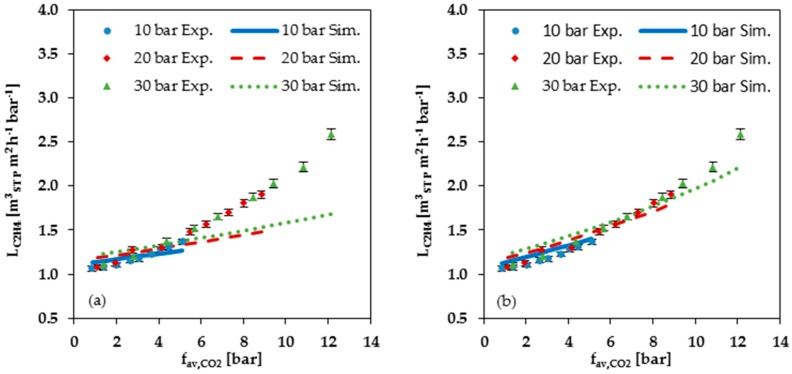

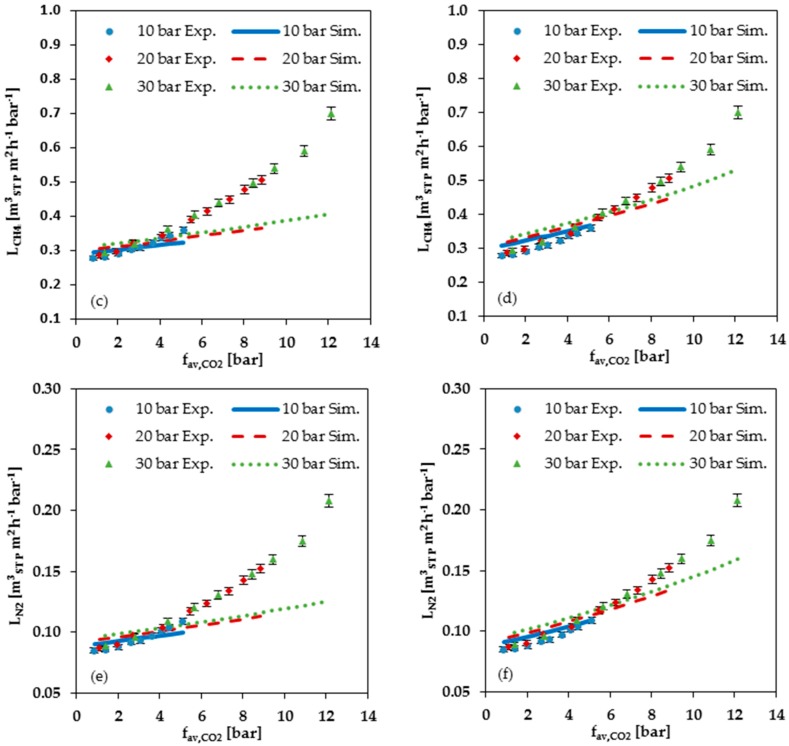
Comparison of measured data (Exp.) with simulated data (Sim.) for C_2_H_4_, CH_4_ and N_2_ permeance at 25 °C plotted against average CO_2_ fugacity: (**a**,**c**,**e**) Simulation data based on FVM parameters determined from low pressure (up to 2 bar) single gas data; (**b**,**d**,**f**) Simulation data based on FVM parameters determined from high pressure (2 to 30 bar) single gas data.

Figure 9 shows a comparison of the measured data with the values calculated with the FVM for C_2_H_4_, CH_4_ and N_2_. As can be seen from this Figure, the FVM underestimates the permeances when the average fugacity becomes larger than 4 bar in cases using the parameters determined at low pressure; see Figure 9a,c,e. In cases using the parameters determined at high pressure, the overestimation starts at an average CO_2_ fugacity of about 6 bar; see Figure 9b,d,f. Up to this pressure, the simulated permeances are higher than the measured ones. Thus, both simulations show an increasing underestimation with rising CO_2_ fugacity for the permemances of the gas mixture components, with the exception of CO_2_ itself. Neither the parameters determined from the low-pressure range, nor the parameters determined from the high-pressure range, provide a sufficient description of gas mixture experiments in cases with average CO_2_ fugacities exceeding 4 to 6 bar. Considering the CO_2_ single gas data (Figure 5a,b), it is apparent that there is no constant CO_2_ swelling parameter which is valid for the entire investigated pressure range. 

In general, the comparison of measured with simulated data show that the FVM cannot accurately predict the permeance at large dissolved quantities of the swelling component in the membrane due to its higher fugacity in the gas phase. The degree of precision of the FVM prediction of permeances for a gas mixture experiments appears to depend on the chosen data set used for the determination of the parameters.

As a consequence of the pronounced swelling of the PolyActive™ thin film composite membrane in CO_2_ containing gas mixtures, the selectivity decreases at higher pressures. In the conducted gas mixture experiments, CO_2_ is mainly responsible for the swelling, and thus, the decrease of selectivity, whereas the swelling influence of C_2_H_4_ is significantly smaller due to both small partial pressure and different interaction between PolyActive™ and C_2_H_4_, as shown for the case of high pressure single gas measurements (Figure 5). In general, the CO_2_/C_2_H_4_ selectivity has small values between 2.4 and 3.4 in the mixed gas experiments, which is usual for this pair of gases, according to data reported in the literature [16]. Additional information regarding the selectivities resulting from the data presented in Figure 9 can be found in the Supplementary Materials. The CO_2_/CH_4_ selectivity has values between 8.7 and 13.2, and the CO_2_/N_2_ selectivity has values between 29.2 and 42.2. These values are also comparable to the selectivity of other polymer membrane materials [16]. Hence, the experimental results of this study show that the PolyActive™ TFCM is suited for CO_2_ separation from a C_2_H_4_ containing gas mixture. However, the FVM can be applied only in the pressure range where the swelling influence of the components in the gas mixture is relatively small.

## 4. Conclusions

The characteristics of PolyActive™ thin film composite membranes were investigated for CO_2_ separation from C_2_H_4_ containing gas mixtures at high pressures. The measurement series for 10-, 20- and 30-bar feed pressure showed no significant dependence of the permeance on the absolute pressure. However, the measurement results proved a strong influence of average CO_2_ fugacity on the permeation characteristics. This phenomenon results from the swelling influence of CO_2_ on the membrane. Hence, the permeances of C_2_H_4_, CH_4_ and N_2_ increase with rising average CO_2_ fugacity. Thus, at high pressures, PolyActive™ membranes show pronounced swelling, depending on the amount of CO_2_ dissolved in the membrane. Up to an average CO_2_ fugacity of 6 bar, the membrane shows a good separation performance for the multi-component gas mixture with C_2_H_4_, which can be predicted with the FVM. With rising average CO_2_ fugacity, the deviation between measured and simulated data increases. Simulations based on parameters determined from single gas data at low and high pressures indicate that the FVM swelling parameter for CO_2_ depends on pressure for the investigation reported here. The fact that the used FVM delivers an inadequate consideration of swelling caused by CO_2_ is in agreement with the findings of a pilot plant project. These findings showed that the FVM is incapable of predicting the membrane separation behavior in case of CO_2_ removal from a C_2_H_4_ containing gas mixture at absolute feed pressures greater than 10 bar [5]. The CO_2_ single gas data shown in the present study indicate a changing swelling parameter of CO_2_ depending on the average fugacity. The pressure range, within which the parameter seems to change, corresponds to the pressure range where the simulation data does not fit with the measurement data. In order to accurately predict the permeances of PolyActive^TM^ membranes at higher feed pressures, a modification of the FVM is required. In further investigations, we want to study whether a modified isotherm consideration or a fugacity dependent swelling parameter can give a better prediction for gas mixture experiments at higher pressures. For the application of the membrane, it is important to consider that the swelling of PolyActive™ membranes leads to a significant decrease in selectivity with increasing average CO_2_ fugacity. Hence, the applicability of the membrane for CO_2_ removal from C_2_H_4_ containing gas mixtures is limited to an average CO_2_ fugacity up to 6 bar. Nevertheless, there are possible applications for a membrane with these characteristics, for example, multistage membrane processes.

## Figures and Tables

**Table 1:** Free Volume Model parameters determined from single gas measurements at 25 °C.

Gas i	$L_{i}^{0}$ $\left[m^{3}{}_{STP}\;m^{-2}h^{-1}bar^{-1}\right]$		$L_{i,average}$ $\left[m^{3}{}_{STP}\;m^{-2}h^{-1}bar^{-1}\right]$		$m_{i}$ $\left[bar^{-1}\right]$		$\sigma_{i}$ [14] $\left[\dot{A}\right]$
Gas i	0–2 bar	2–30 bar	0–2 bar	2–30 bar	0–2 bar	2–30 bar	
CO_2_	3.29 ± 0.01	2.97 ± 0.15	-	-	0.0352	0.0572	3.94
C_2_H_4_	1.04 ± 0.01	1.01 ± 0.04	-	-	0.0470	0.0470	4.16
CH_4_	-	-	0.274 ± 0.002	0.281 ± 0.006	-	-	3.76
N_2_	-	-	0.084 ± 0.001	0.083 ± 0.002	-	-	3.80

## Supplementary Materials

The following are available online at http://www.mdpi.com/2077-0375/8/2/27/s1, Figure S1: CO_2_/C_2_H_4_, CO_2_/CH_4_ and CO_2_/N_2_ selectivity at 25 °C plotted against average CO_2_ fugacity from mixed gas experiments, Figure S2: Position of the gas transport properties of PolyActive™ among those of polymers reported in the literature.
