# Supplementary material for: Applicability of PolyActive™ Thin Film Composite Membranes for CO2 Separation from C2H4 Containing Multi-Component Gas Mixtures at Pressures up to 30 Bar
Source: Membranes (Basel). 2018 Jun 5;8(2):27. doi: 10.3390/membranes8020027 (PMC6027195; doi:10.3390/membranes8020027)
Supplement: Supplementary file 1 [file membranes-08-00027-s001.pdf]

Supplementary Materials: Applicability of PolyActive™ Thin Film Composite Membranes for CO_2 Separation from C_2H_4 containing Multi-component Gas Mixtures at Pressures up to 30 bar

Karina Schuldt, Jan Pohlmann, Sergey Shishatskiy and Torsten Brinkmann

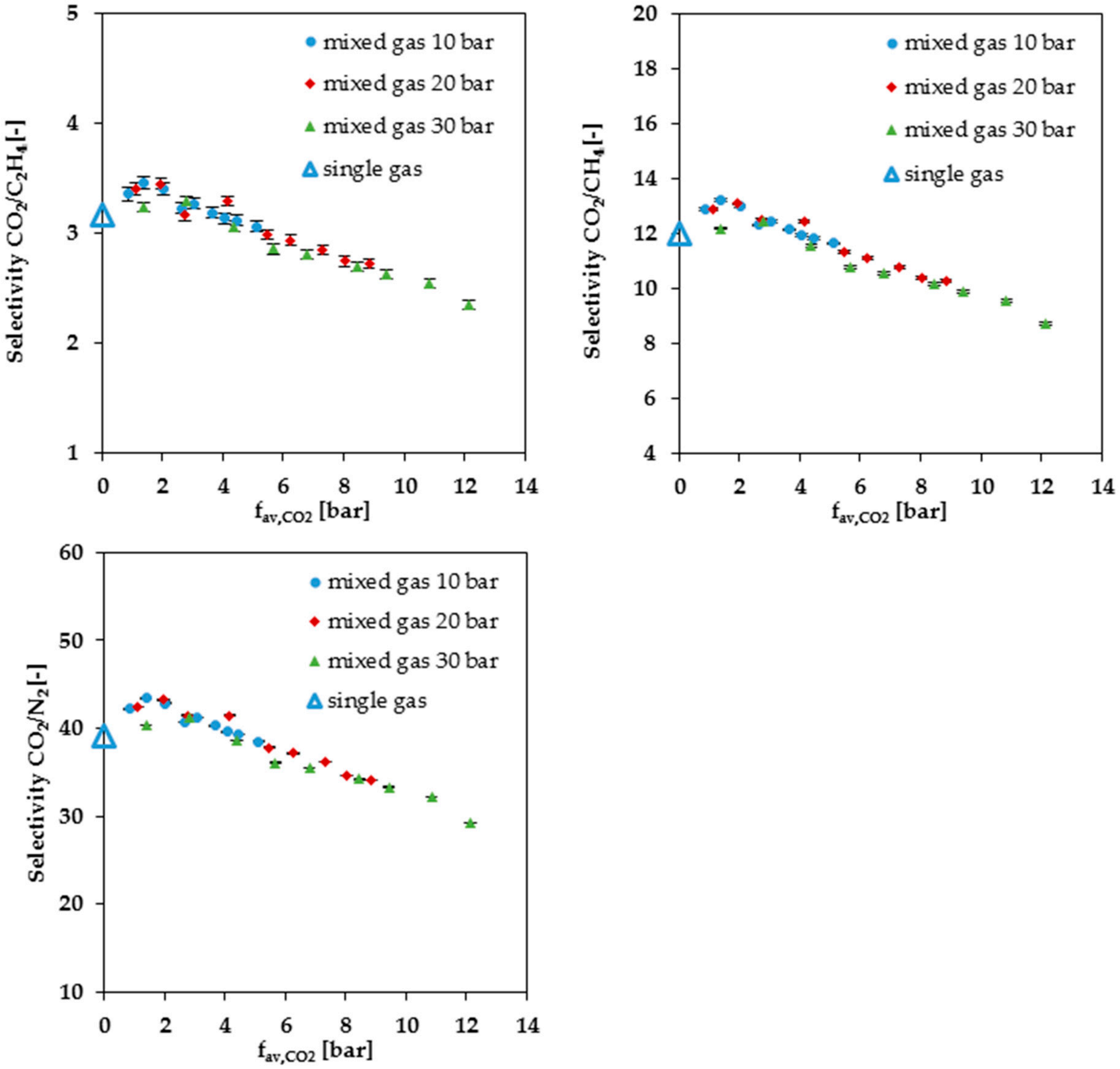

Figure S1. CO_2/C_2H_4, CO_2/CH_4 and CO_2/N_2 selectivity at 25 °C plotted against average CO_2 fugacity from mixed gas experiments. The open triangle represents single gas data acquired at low pressure (0–2 bar), i.e. pressure increase data where the plotted value was determined using the L_0 values of the Free-Volume model according to Equation (1). In this study, the selectivity is defined as the ratio of permeances.

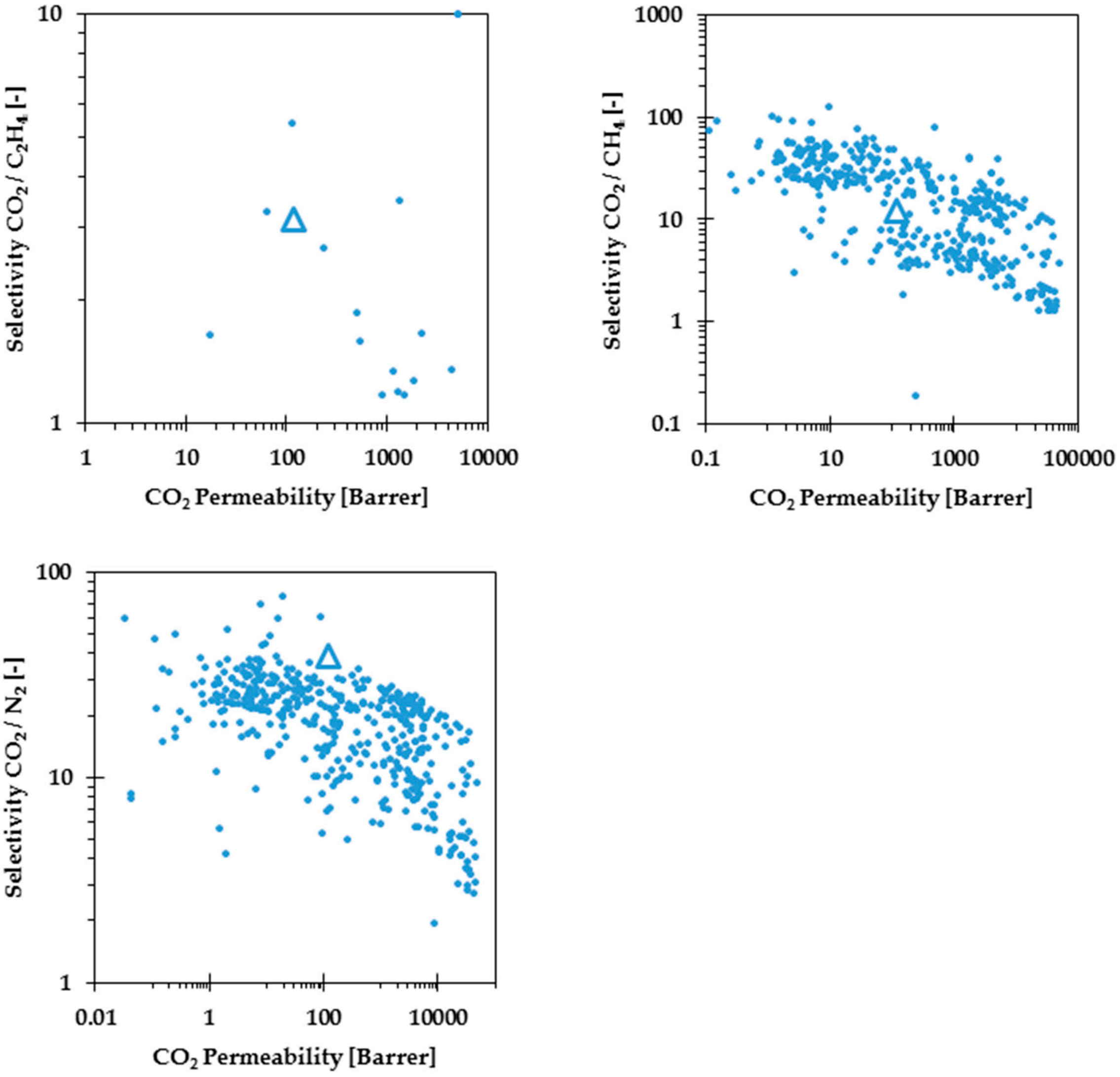

Figure S2. Position of the gas transport properties of PolyActive ™ among those of polymers reported in the literature [1]. PolyActive™ is marked by open triangle.

Reference

1. Polymer gas separation membranes. Available online: https://membrane-australasia.org/polymer-gas-separation-membranes/ (Accessed on 29 May 2018)
